# High-Performance Iontronic Hydrogel Acoustic Sensor for Low-Frequency Underwater Sound Detection and Intelligent Recognition

**DOI:** 10.34133/research.1292

**Published:** 2026-05-28

**Authors:** Jiawei Zhao, Honglei Zhou, Tongqiang Fu, Haijun Wang, Tangjia Zhang, Longqiu Li

**Affiliations:** ^1^ Zhengzhou Advanced Research Institute of Harbin Institute of Technology, Zhengzhou 450000, China.; ^2^ State Key Laboratory of Robotics and System, Harbin Institute of Technology, Harbin 150001, China.; ^3^ School of Mechanical Engineering, Xi’an Jiaotong University, Xi’an 710049, China.

## Abstract

The detection and identification of low-frequency underwater sounds remain challenging due to inefficient acoustic–mechanical–electrical coupling, which limits the conversion of low-frequency weak pressure fluctuations into stable electrical signals in conventional hydrophones. Here, we develop a capacitive hydrogel acoustic sensor (CHAS) that enhances low-frequency transduction through a micro-pyramid iontronic structure governed by dynamic electric double layer (EDL) effects. By combining the EDL-based sensing mechanism with a phase-sensitive lock-in amplification circuit providing a 40 dB gain, the system preserves weak acoustic information within the 20 to 1,000 Hz range. The sensor achieves an average sensitivity of −158.86 dB and reliably detects diverse low-frequency underwater acoustic events, including human speech, water impacts, and vessel-radiated noise. A neural network trained on the ShipsEar database yields 96.3% accuracy and, especially, maintains 89.1% accuracy when directly applied to CHAS-acquired signals without retraining, demonstrating that the iontronic sensing mechanism preserves physically meaningful acoustic features and enables reliable cross-domain intelligent analysis. This work establishes an integrated sensing framework that links iontronic device physics, circuit-level signal conditioning, and data-driven acoustic interpretation, providing a practical pathway toward intelligent low-frequency underwater acoustic monitoring and target recognition.

## Introduction

Low-frequency (<1,000 Hz) underwater acoustic sensing plays a critical role in a wide range of ocean-related activities, including marine resource exploration, environmental monitoring, underwater navigation, and maritime security [1–4]. In practical ocean environments, especially in shallow-water or high-noise conditions, weak low-frequency acoustic signals are strongly affected by ambient noise and multipath propagation, making reliable detection and identification particularly challenging. Although various underwater acoustic sensors such as piezoelectric ceramic hydrophones, polyvinylidene fluoride hydrophones, and micro-electro-mechanical system hydrophones have been developed, low-frequency signal detection remains difficult due to limited sensitivity and noise interference [5–9]. These limitations arise not only from material sensitivity but also from the reduced efficiency of acoustic–mechanical–electrical coupling at low frequencies, where small pressure variations are difficult to convert into stable electrical signals. As a result, improving low-frequency underwater acoustic sensing requires not only enhanced sensing materials but also new transduction and system-level strategies capable of reliably extracting weak acoustic information.

Hydrogel-based acoustic sensors have recently attracted considerable attention owing to their mechanical softness and high water content, which provide acoustic impedance matching with water [10,11]. Under acoustic pressure, interfacial electrochemical interactions within the hydrogel produce measurable electrical responses [12]. At the hydrogel–electrode interface, the electric double layer (EDL) enables efficient interfacial charge accumulation through ion–electron coupling [13,14]. Mechanical deformation induced by acoustic pressure modifies the ionic distribution and interfacial contact area, leading to substantial modulation of EDL capacitance [15–18]. Unlike conventional dielectric capacitive sensors, EDL-based iontronic sensors operate through nanoscale interfacial charge separation, providing enhanced sensitivity to small mechanical perturbations [19–21]. These characteristics provide an effective physical pathway for enhancing low-frequency acoustic transduction efficiency [22–24]. Various strategies have been explored to improve hydrogel sensor sensitivity. Gao et al. [25] used silver nanotrees in deformed hydrogels to generate capacitance signals for sound wave detection. Li et al. [26] combined a hydrogel with monolayer graphene, utilizing dynamic EDL interactions within graphene sheets to achieve low-frequency acoustic sensing in the 20 to 200 Hz range with a sensitivity of approximately −200 dB. Meng et al. [27] created a wooden hydrophone with conductive wooden microbranches as electrodes and a hydrogel as a dielectric layer, achieving −174 dB sensitivity within the frequency range of 250 to 2,300 Hz. Despite these advances, achieving simultaneously high sensitivity and stable response in the low-frequency regime remains a considerable challenge. In realistic underwater environments, weak acoustic pressures, interfacial instability, and environmental perturbations often induce sensitivity fluctuations and reduce signal reliability. Furthermore, many reported devices still rely on simplified structures or complicated fabrication processes, which can introduce instability and hinder reliable acoustic interpretation and intelligent analysis in realistic underwater environments.

The above limitations indicate that material optimization alone is insufficient for practical underwater acoustic sensing. Real-world applications require not only sensitive detection but also reliable signal conditioning and intelligent interpretation capabilities. While sensor sensitivity determines detection limits, effective identification of underwater targets ultimately depends on robust signal analysis and classification algorithms [28,29]. Traditional signal processing approaches, such as Fourier or wavelet-based feature extraction, capture basic spectral characteristics but often fail to fully exploit the underlying relationships between radiated noise sources and sensor responses [30,31]. Machine learning methods offer powerful tools for extracting latent time–frequency features and improving target recognition performance. A fundamental yet often overlooked challenge, however, lies in the cross-domain generalization gap: data-driven models trained on benchmark databases often lose effectiveness when applied to signals collected by real-world sensors with different physical transduction mechanisms [32,33]. Even under identical acoustic excitation conditions, discrepancies in transduction mechanisms and interfacial coupling can fundamentally alter signal feature distributions, causing database-trained models to fail when deployed on real sensors.

Here, we developed an underwater capacitive hydrogel acoustic sensor (CHAS) for highly sensitive detection of low-frequency sound signals and constructed an adaptive classification model for oceanic targets. By employing a hydrogel micro-pyramid array design, CHAS enhances interfacial deformation and EDL modulation under acoustic pressure, thereby improving the electromechanical coupling efficiency. Complementary to our earlier work on material-level sensitivity enhancement through structural engineering [23], the present study emphasizes system-level optimization. A lock-in amplification circuit with a 40-dB electrical gain was integrated to boost signal detectability and extend the effective frequency response range to 20 to 1,000 Hz. Through transient EDL modulation, the sensor achieves an average sensitivity of −158.86 dB and exhibits promising performance in detecting weak low-frequency underwater signals, such as human voices and splash sounds, which are representative acoustic events in applications such as underwater rescue, diver monitoring, and human activity detection. Moreover, the sensor effectively captured radiated noise from multiple marine vessels, demonstrating its practical capability in underwater acoustic monitoring. In parallel, a low-frequency target classification model was established using a multilayer perceptron (MLP) algorithm, achieving 96.3% recognition accuracy on a reference database and 89.1% on CHAS-acquired signals. The successful transfer of the classification model from database data to real-world measurements underscores the robustness and generalizability of the approach. Overall, this work advances hydrogel-based capacitive acoustic sensors from material-level design toward integrated circuit–algorithm co-optimization, positioning the proposed acoustic sensor as a promising platform for low-frequency underwater detection and intelligent acoustic sensing.

## Results and Discussion

### Design and fabrication of CHAS

**Fig. 1.**
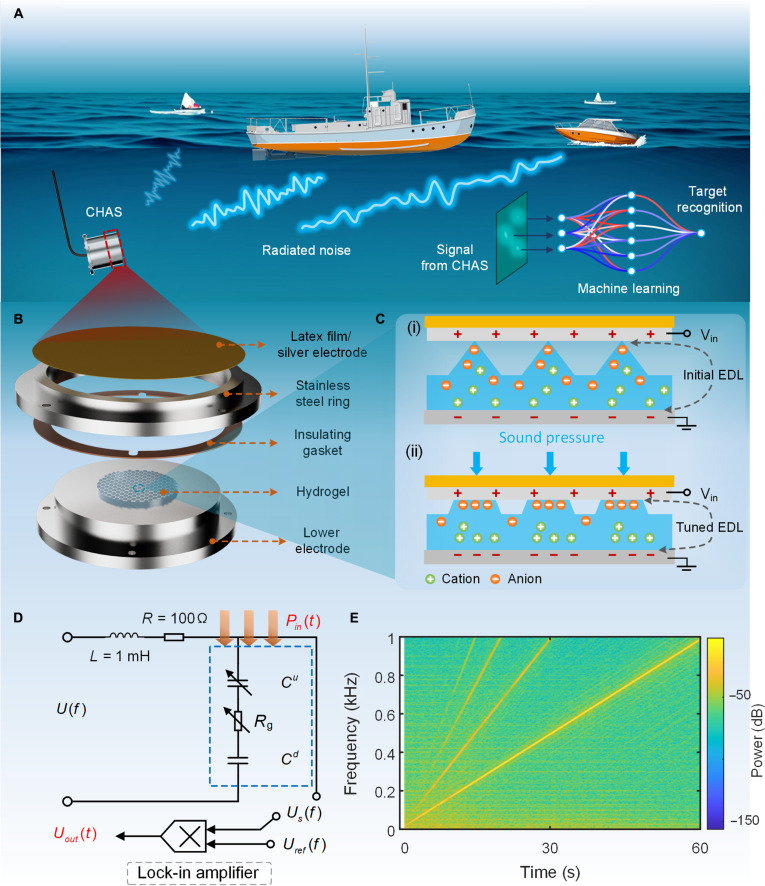
System overview and working principle of the capacitive hydrogel acoustic sensor (CHAS). (A) The sensor aims to monitor low-frequency dynamic signals related to underwater targets and classify them. (B) Exploded view of the multilayer structure of the sensor. (C) Schematic of the transduction mechanism in the sensor when sensing sound pressure. (D) The signal processing circuit of the sensor. (The blue dashed area represents the sensor; *P_in_*(*t*) represents the sound wave signal, and *U_out_*(*t*) represents the circuit output signal.) (E) Frequency spectrum of the sensor toward underwater acoustic signals from 20 to 1,000 Hz.

Figure 1A illustrates the scenario in which the proposed acoustic sensor is applied. This sensor can detect low-frequency underwater targets, such as ships moving on the surface. Based on this, we developed a machine-learning-based signal classification model to efficiently and accurately identify targets from the acquired signals. This approach extends the application scope of the sensor and verifies its practical feasibility through subsequent experiments. For accurate measurements, the sensor is installed in an aluminum alloy cylinder, and signals are transmitted via a waterproof cable. Figure 1B shows the core components of the sensor, including a latex film/silver electrode, a stainless steel ring, an acrylamide (AAm) hydrogel, a stainless steel electrode, and an insulating gasket. The primary function of the insulating gasket is to prevent contact between the upper and lower electrodes while providing support to reduce initial pre-pressure on the sensing material. Figure 1C illustrates the sensing mechanism of the sensor. An ac bias is continuously applied to the device during operation, establishing an interfacial electric field at the electrode–hydrogel interface and forming an initial EDL under electrochemical equilibrium conditions. When the electrolyte composition is determined, the capacitance response rate of EDL-based capacitive sensor mainly correlates with the rate of change in the interface area. The capacitance of CHAS is primarily generated by the contact between the electrode and the tip of the pyramid-shaped gel, as well as the base of the gel. In this study, the capacitance generated at the gel base remains relatively constant; therefore, the sensing response mainly originates from the pyramid gel tip. When no external pressure is applied, the pyramid microstructure contacts the electrode only at its tip, resulting in a limited interfacial area and a relatively small initial capacitance (Fig. 1C (i)). When acoustic pressure is applied, the pyramid microstructure undergoes compression and deformation, increasing the electrode–gel contact area. Meanwhile, the pre-established interfacial electric field drives the redistribution of mobile ions within the hydrogel. Oppositely charged ions migrate toward the electrode surface to continuously maintain electrochemical equilibrium, leading to dynamic reconstruction of the EDL. The coupled processes of mechanical deformation and ion migration produce pronounced modulation of interfacial charge density and EDL capacitance (Fig. 1C (ii)). Importantly, unlike resonance-based piezoelectric transduction, the iontronic sensing mechanism relies on rapid and dynamically reversible interfacial charge redistribution rather than mechanical resonance. Because ionic migration and EDL relaxation occur on a time scale much shorter than the period of low-frequency acoustic excitation, enabling stable sensing even at frequencies as low as 20 Hz. The theoretical relationship between the capacitance and the gel/electrode contact area can be obtained in Note S1. Unlike our previous carbon-nanotube-doped hydrogel design [23], where enhanced charge transport improved sensitivity at the material level, the present CHAS employs a pure AAm hydrogel. Although the generated charge is smaller without conductive fillers, the pure hydrogel ensures superior dielectric uniformity and interfacial stability, effectively preventing local field concentration and micro-breakdown effects. To compensate for the reduced intrinsic signal level, a lock-in amplification circuit with a 40-dB electrical gain was integrated into the system, enabling effective detection of weak low-frequency acoustic signals.

To convert the capacitance signal into a voltage signal, we designed a dedicated conversion circuit module comprising a voltage divider circuit and a phase-locked module (Fig. 1D). The sensor can be modeled as 2 sets of EDL capacitors in series with a bulk resistor, where the equivalent circuit of the sensor is represented by the blue dashed area. The sensor is excited with an ac voltage (*U*_f_: 20 mV, 40 kHz), and the voltage signal across it, processed through the phase-locked filtering module (*U*_ref_: 1 V, 40 kHz), ultimately provides the output representing the low-frequency underwater acoustic signal (*U_out_*(*t*)). Through rational structural and circuit design, the sensor exhibits a clear frequency response to low-frequency sound signals (*P_in_*(*t*)), with the spectrum in the range of 20 to 1,000 Hz shown in Fig. 1E.

**Fig. 2.**
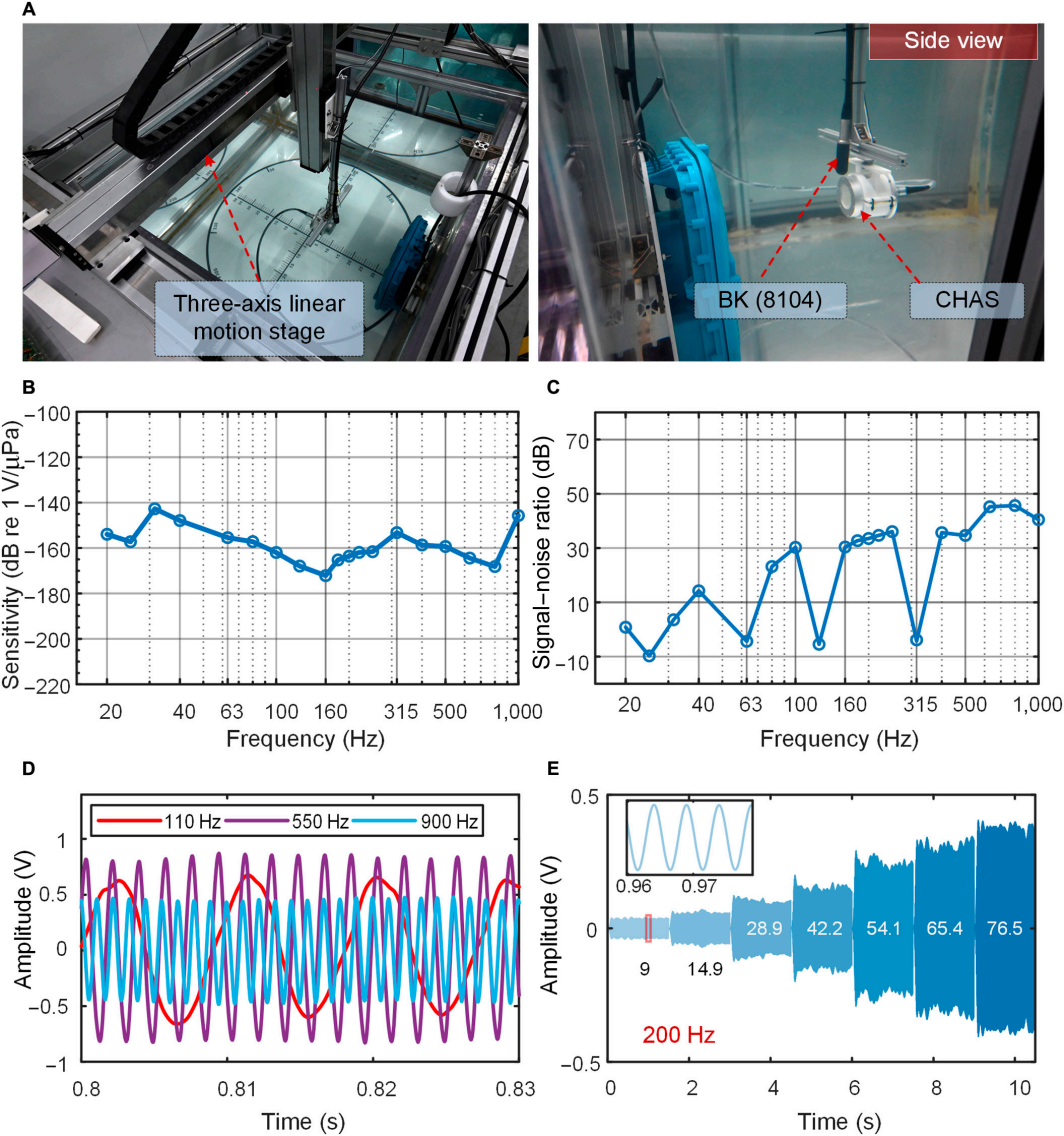
Response of the capacitive hydrogel acoustic sensor (CHAS) to underwater acoustic waves. (A) Experimental scenario of CHAS. (B) Frequency response ranging from 20 to 1,000 Hz. (C) Signal-to-noise ratio (SNR) within the 20- to 1,000-Hz range. (D) Time-domain response of CHAS under varying sound frequencies. (E) Time-domain response at different sound pressure levels at 200 Hz. The inset shows the time-domain waveform when the sound pressure is 9 Pa.

We tested the performance of CHAS in an acrylic water tank (Fig. 2A). CHAS and the standard hydrophone BK (8104) were placed on the acoustic axis of an underwater loudspeaker, with BK (8104) used to calibrate the sound pressure at the measurement point. The sensitivity grade of the hydrophone can be expressed as $L_{p}=20\mathrm{lg}\left(U_{p}/U_{\mathrm{ref}}\right)+L_{\mathrm{ref}}$ [34], where *L_p_* is the sensitivity grade of the measured hydrophone; *U_p_* and *U_ref_* are the output voltages of the measured hydrophone and the standard hydrophone, respectively; and *L_ref_* is the system sensitivity of the standard hydrophone after the conditioning amplifier (−160 dB re 1 V/μPa). Note that the output voltage of CHAS was amplified with a gain of 40 dB using a signal-conditioning circuit before performing the sensitivity calculation.

By emitting discrete sinusoidal signals of specific frequencies from the underwater loudspeaker, we used CHAS to receive and record them, thus obtaining its frequency response curve (Fig. 2B). At room temperature, the sensor exhibited high sensitivity, with an average sound pressure sensitivity of −158.86 ± 15 dB and a bandwidth of 20 to 1,000 Hz. Table 1 lists a comparison of various reported hydrophones with this work. The hydrophone developed in this study demonstrates superior sensitivity in the low-frequency range compared with previously reported hydrophones. In terms of the operating frequency range, high-frequency sound waves attenuate rapidly underwater, while the sensor covers most of the low-frequency target radiation noise bands, enabling it to detect distant targets. Signal-to-noise ratio (SNR) is another important metric for evaluating the performance of underwater acoustic sensors. It is generally expressed as $\mathrm{SNR}=10\mathrm{lg}\left(P_{s}/P_{n}\right)$, where *P_s_* represents the average power of the signal and *P_n_* represents the average power of the noise. Figure 2C illustrates the SNR values of CHAS within the frequency range of 20 to 1,000 Hz. Except for several low-frequency points where the SNR falls below 10 dB, most measured signals exhibit SNR values exceeding 20 dB. Particularly, at frequencies of 100 Hz and above, the SNR approaches or even exceeds 30 dB, indicating that the impact of noise on the signal is minimal and negligible.

**Table 1.** Comparisons of various reported underwater acoustic sensors with this work

Sensing mechanism	Transducing material	Sensitivity (re 1 V/μPa)	Working frequency range	Ref.
Capacitive	/	−171 dB at 1 kHz ^a^	20–1,000 Hz	[7]
Capacitive	Hydrogel	−174 dB	20–800 Hz	[27]
Triboelectric	Polytetrafluoroethylene film	−171 dB at 220 kHz	150–500 Hz	[40]
Fiber optic	Multimode microstructured fiber	−213 dB	100–10,000 Hz	[41]
Piezoelectric	PVDF	−211 dB at 500 Hz	20–500 Hz	[6]
Capacitive	Hydrogel	−158.86 dB	20–1,000 Hz	This work

PVDF, polyvinylidene fluoride

^a^ 40 dB amplified.

We compared the time-domain output voltage signals of CHAS at 110, 550, and 900 Hz (Fig. 2D). The acoustic signals detected by the sensor exhibit low noise levels and clear waveform characteristics. In particular, the sensor is sensitive to low-frequency sound waves and exhibits a marked voltage response. This is primarily due to the unique EDL sensing mechanism and conditioning circuit of the sensor. Figure 2E demonstrates the voltage response of the sensor at specific frequencies (200 Hz) under different sound pressures. Data processing involves employing a ±20 Hz band-pass filter to remove interference from the experimental environment. As shown in Fig. 2E, the output voltage of the sensor increases with increasing sound pressure. It is noteworthy that even at sound pressures as low as 9 Pa, clear and stable waveforms can be observed. Based on the same set of measurements shown in Fig. 2E, the output voltage of the sensor is plotted against the input sound pressure at 200 Hz to evaluate its linearity. A linear fit yields a slope of 0.0049 V/Pa with an *R*^2^ of 0.98, indicating good linearity within the tested range (Fig. S1).

### Response of CHAS to various acoustic stimuli

**Fig. 3.**
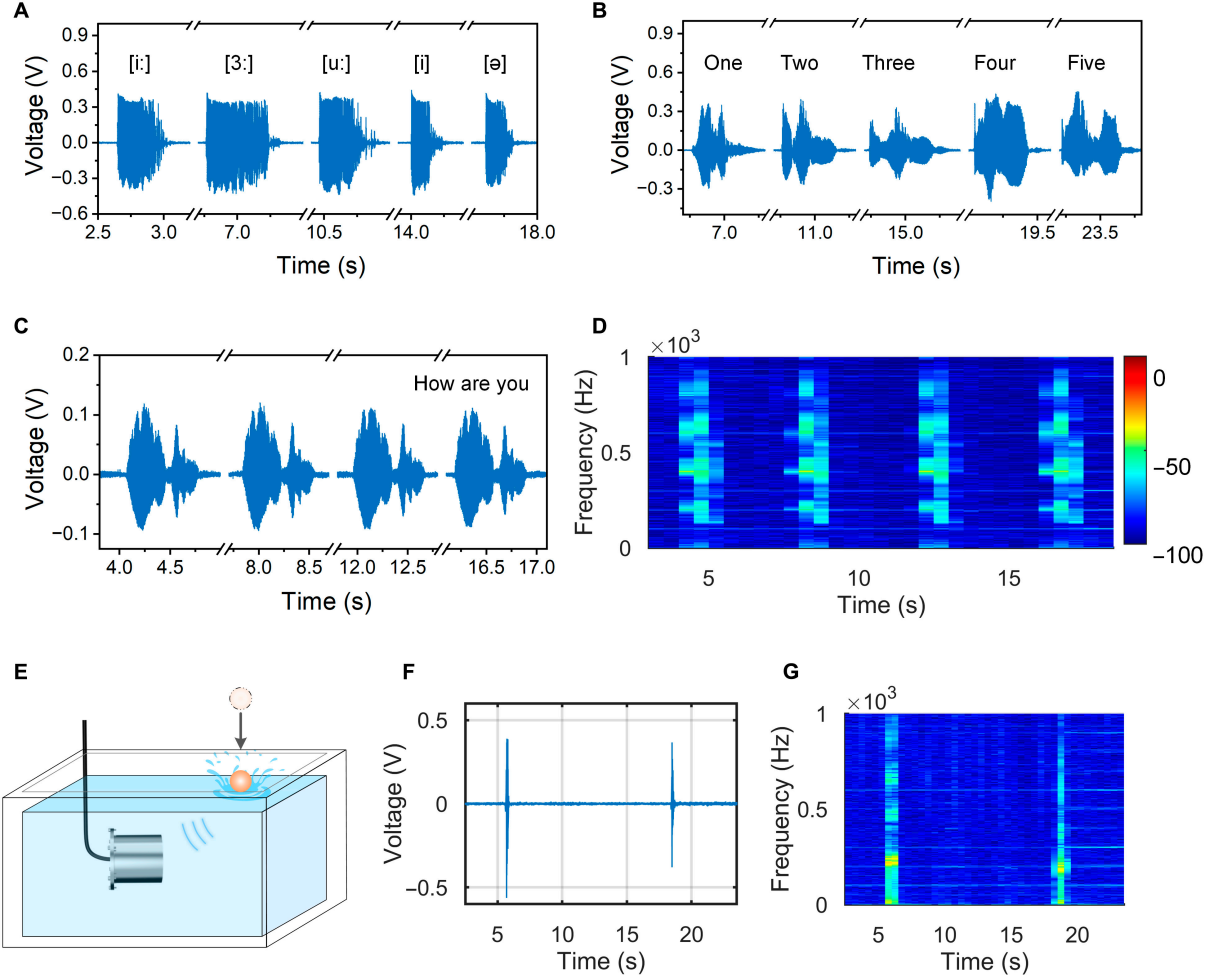
Responses of capacitive hydrogel acoustic sensor (CHAS) to various acoustic stimuli and vibration detection demonstrations. (A) CHAS’s response to different vowels. (B) Response to different words. (C) Response to 4 repeated pronunciations of the sentence “How are you”. (D) Spectrogram of the audio “How are you”. (E) A demonstration setup showing CHAS detecting both acoustic and vibrational waves caused by a resin block free-falling into water. (F) Output voltage when the falling resin block strikes the water’s surface. (G) Spectrogram of the voltage signal.

Generally, the frequency range of human speech is from about 50 Hz to 8 kHz [35,36]. The low-frequency components (50 to 300 Hz) in speech signals are typically used to convey emphasis, focus, and emotional aspects of language, while the high-frequency components (300 Hz to 8 kHz) are used to convey detailed information about speech articulation. Figure 3 illustrates the perception results of CHAS for 2 scenarios, underwater human speech and objects falling into the water, demonstrating its outstanding sensitivity and stability in responding to low-frequency sounds. Figure 3A shows that CHAS can effectively capture and distinguish differences between phonemes. The voltage response signals representing different phonemes exhibit varying amplitudes and characteristic shapes. Additionally, we tested 5 simple words, “one”, “two”, “three”, “four”, and “five”, revealing distinct differences in the voltage response signal characteristics (Fig. 3B). Figure 3C depicts the time-domain waveforms of the repeated measurements of the sentence “How are you” 5 times, demonstrating good repeatability in voltage changes and characteristic peaks under the same pronunciation. Figure 3D displays the short-time Fourier transform (STFT) spectrogram of the sentence “How are you”, repeated 5 times. The consistent patterns in the spectrogram suggest that CHAS’s response waveforms exhibit good synchronization. This is attributed to its high sensitivity, enabling stable real-time response to sound signals.

Lastly, a spherical object with a diameter of 40 mm was dropped from a height of 40 cm above the water surface (Fig. 3E). CHAS detected the acoustic and vibrational waves generated by the impact on the water surface, with the output voltage shown in Fig. 3F. A 15 to 1,100 Hz band-pass filter was applied to the raw signal to suppress low-frequency hydrodynamic noise while retaining the primary acoustic and vibrational features. Due to energy dissipation from damping motion, the received signal waveform exhibited oscillation and attenuation, with the maximum voltage amplitude reaching 745 mV (Fig. S2). Analysis of the voltage signals collected by CHAS using STFT revealed that the primary energy characteristics of the signal were concentrated around 20 and 200 Hz (Fig. 3G). Notably, the energy distribution of the signal ranged from 0 to 1,000 Hz, demonstrating CHAS’s ability to detect signals with frequencies up to 1,000 Hz. Additionally, we used CHAS to record a segment of the music “China-E”, further validating its ability to accurately capture complex low-frequency signals (Fig. S3 and Movie S1). By utilizing different types and positions of sound sources, we demonstrated CHAS’s outstanding sensitivity and distinctiveness in detecting low-frequency sound signals. These experimental results further validate the considerable potential of the novel sensor in underwater acoustic detection.

**Fig. 4.**
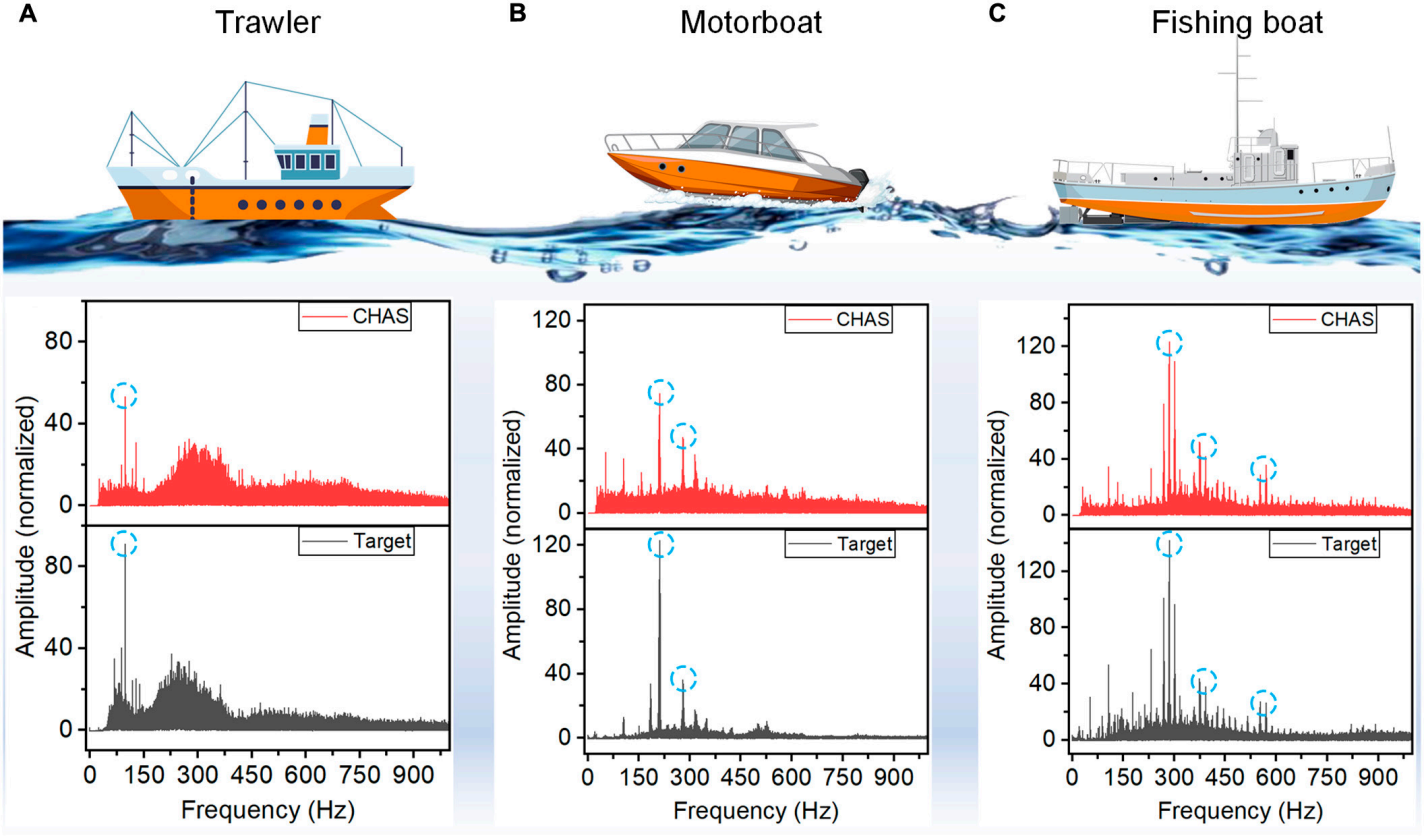
Detection of different marine vessel noise signals. (A) Trawler. (B) Motorboat. (C) Fishing boat.

To demonstrate the practical application of CHAS in underwater target identification, we conducted experiments to detect the radiated noise of different maritime vessels. The noise signals from a fishing boat, a trawler, and a motorboat were sourced from the ShipsEar database (accessible at http://atlanttic.uvigo.es/underwaternoise/). In a controlled environment, a speaker was used to simulate the distinctive frequencies and signal intensities of these targets. Figure 4 illustrates the spectrograms of the 3 vessel targets and the spectral features detected by the sensor, with normalization applied for a more intuitive comparison. It is evident that CHAS effectively captured the target signals, and the spectral characteristics highly matched those of the targets. The successful detection of low-frequency underwater targets is attributed to the high sensitivity of the sensor, which primarily result from the tuned EDL sensing mechanism facilitated by the micro-pyramid array structure.

### Establishment of a classification model suitable for CHAS

**Fig. 5.**
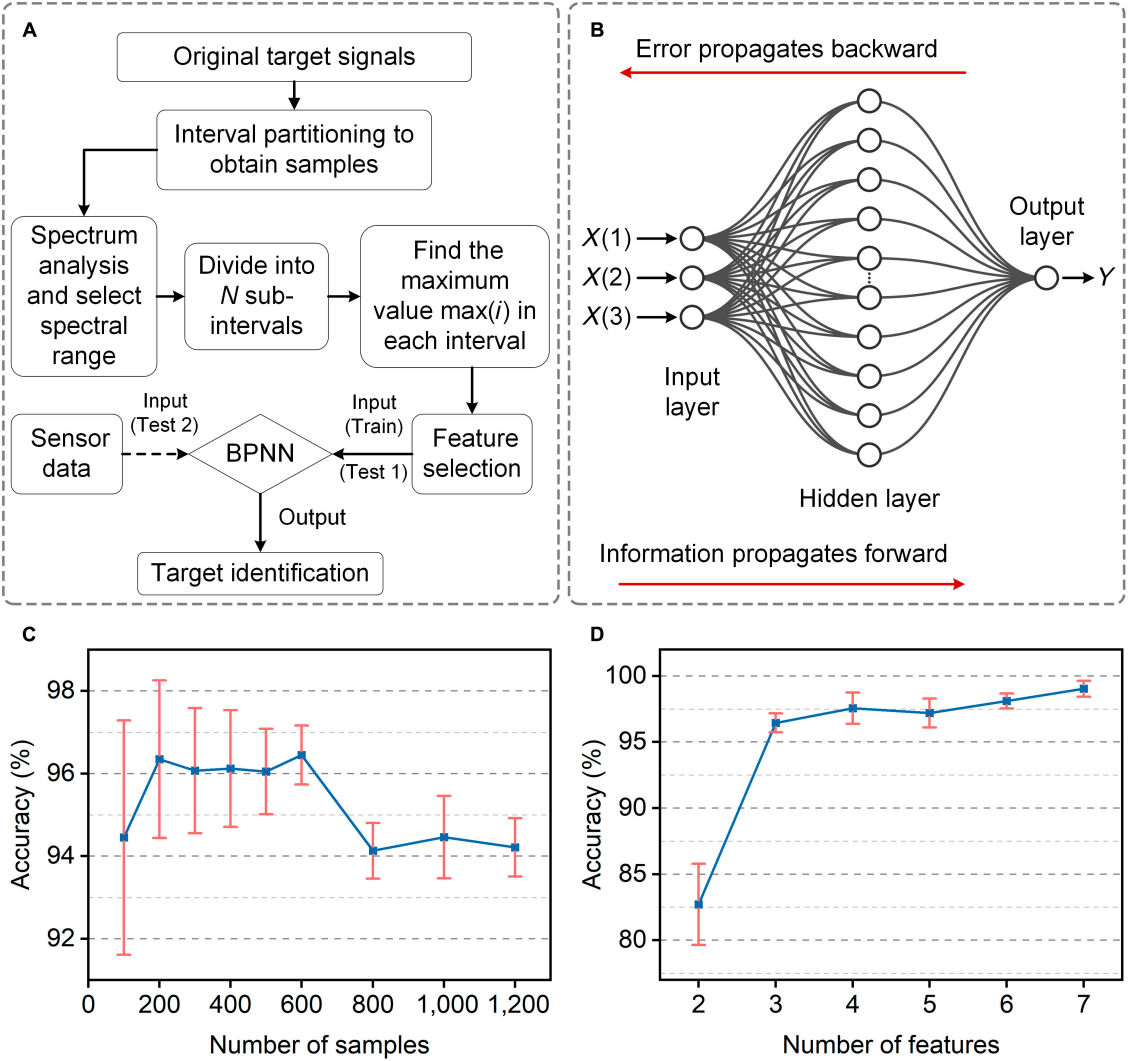
Overview of the backpropagation neural network (BPNN)-based classification analysis. (A) The flowchart for the classification analysis algorithm based on BPNN. (B) Topological structure of the BPNN. (C) The impact of sample size on the accuracy of the classification model. (D) The impact of the number of features on the accuracy of the classification model.

As underwater applications continue to expand, the demand for accurate and reliable underwater target detection and classification systems becomes increasingly urgent. Combining CHAS with machine learning models can provide efficient and accurate solutions for underwater target detection and classification systems. Although the sensor has high sensitivity and good SNR performance, the powerful classification ability of machine learning models is crucial for accurately classifying complex underwater signals. As an emerging technology in the field of machine learning, machine learning can efficiently extract features by learning the inherent patterns and correlations of sample data, thereby achieving accurate predictions of results [37–39]. Therefore, to further validate the potential application of the sensor as an underwater target identification system, we constructed a machine learning classification network using the source data from the database and inputted the signals collected by CHAS into the network for classification recognition. Figure 5A illustrates the process of the backpropagation neural networks (BPNNs) used for the classification task, which includes 4 steps:

Step 1: Sample collection. Each vessel signal is evenly divided into *K* segments (*K* = 600) to obtain a sample set.

Step 2: Feature extraction via spectrogram. Specifically, perform spectral analysis on the original samples, divide the spectrum into *N* intervals, find the maximum spectral peak value in each interval, and establish the sample feature set *M*.

Step 3: Establishing the classification model. Utilize the “Neural Net Pattern Recognition” app in the MATLAB software’s Deep Learning Toolbox to train and test the sample feature data. The training was set to a maximum of 1,000 epochs, with a validation patience of 6 (training stops if the validation error does not decrease for 6 consecutive iterations). The initial learning rate was 0.01, automatically adjusted by the algorithm. The performance goal (mean squared error) was set to 0. The data partition (70% for training, 15% for validation, and 15% for testing) was performed randomly using the default MATLAB function.

Step 4: Repeat steps 1 and 2 to obtain a sample feature set of the underwater acoustic signals collected by the hydrogel underwater acoustic sensor. Then, input this feature set into the classification model to test its classification performance.

Artificial neural networks are extensively utilized for modeling the relationship between inputs and outputs, demonstrating strong performance in tasks such as data classification, inference, and pattern recognition. A commonly used type of neural network within the realm of BPNNs is the MLP. An MLP employs forward and backward propagation algorithms to minimize the mean squared error between the actual and expected outputs via gradient descent. It typically consists of an input layer, one or more hidden layers, and an output layer, with each layer containing multiple nodes. In this study, the MLP uses the sigmoid function as its activation function and incorporates L2 regularization (weight decay). We constructed a 3-layer MLP network, as depicted in Fig. 5B. The input layer consists of 3 nodes, corresponding to the 3 features extracted per sample in step 2. The hidden layer contains 10 nodes, which was determined through empirical trial and error: we tested hidden layer sizes ranging from 5 to 20 and found that 10 nodes yielded the best balance between classification accuracy and generalization.

The size of the sample is closely related to the generalization ability of the classification model. Typically, increasing the number of samples can improve the generalization ability of the classification model, while too few samples may lead to model overfitting. Figure 5C illustrates the relationship between the number of samples and the accuracy of the classification model. Here, we first set the number of features for each sample to be 3, and the accuracy corresponding to each number of samples is the average after 10 repetitions. As the number of samples increases, the classification accuracy first improves and subsequently decreases, indicating a trade-off between training sufficiency and model generalization. When the number of samples increases from 100 to 600, the accuracy rises from 94.45% to over 96%. As the number of samples increases, the error bars become narrower, indicating more consistent performance for the model on this dataset, suggesting better generalization. However, when the sample size continues to increase, the accuracy suddenly drops. This could be due to a larger number of samples containing substantial noise or irrelevant information, which interferes with the model’s ability to recognize and classify target signal features, leading to a decrease in accuracy. Therefore, we decided to set the final number of samples at 600.

The performance of the classification model is not only influenced by the number of samples but also often affected by the number of features. Figure 5D illustrates the relationship between the number of features and the accuracy of the classification model. Accuracy increases with the number of selected features; when only 2 features are used, the classification accuracy remains relatively low at 82.7%. This is because too few sample features may result in the loss of some important information, affecting the performance of the model. As the number of sample features gradually increases to 7, the classification accuracy reaches its highest point, at 99%. It can be seen that increasing the number of features helps the model better capture the complex relationships and characteristics of the data, thereby improving accuracy. However, too many features may introduce noise and unnecessary information, leading to model overfitting. Thus, we choose the final number of sample features to be 3.

**Fig. 6.**
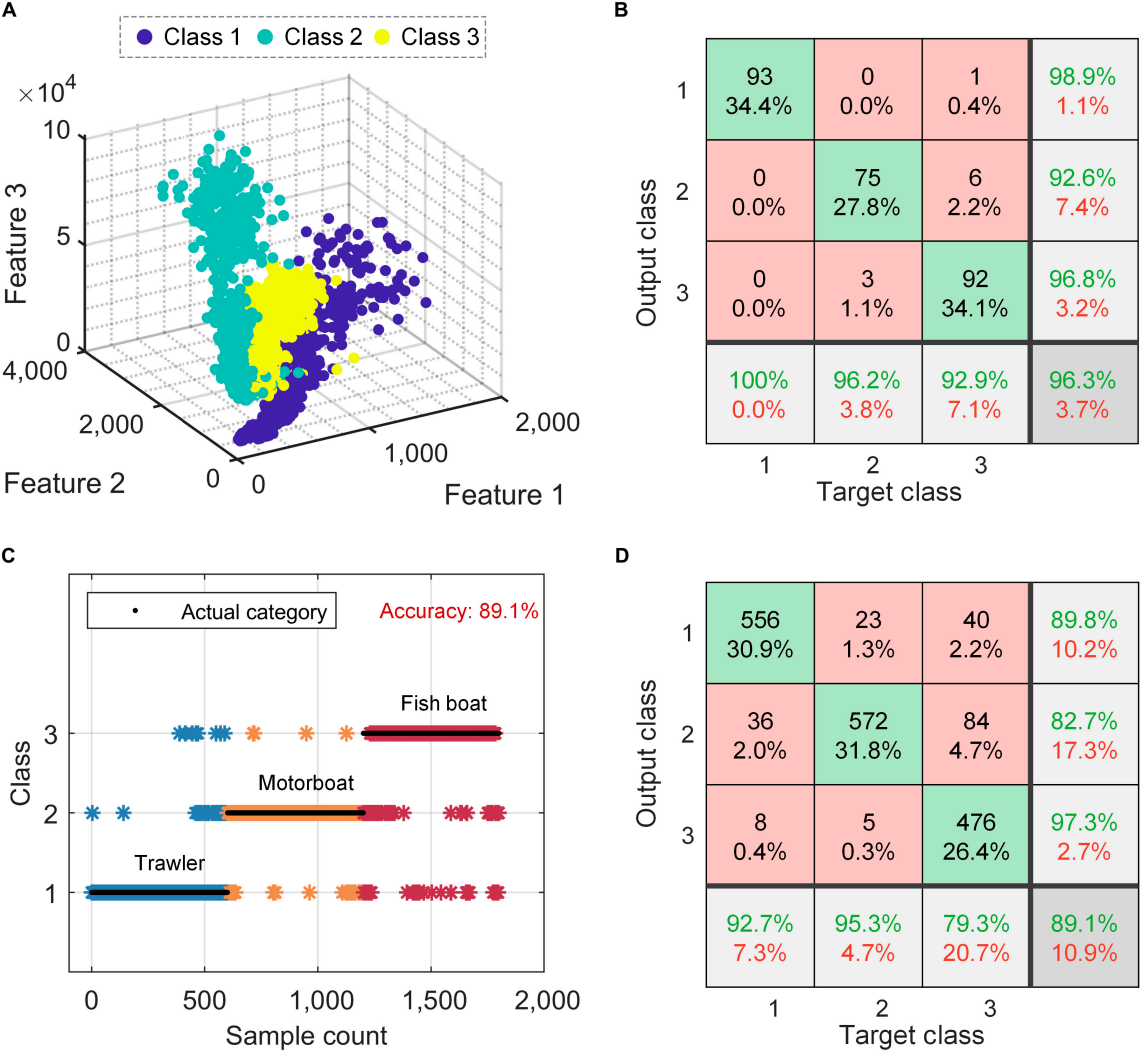
Results and visualization of classification analysis for vessel data and capacitive hydrogel acoustic sensor (CHAS) test data. (A) Visualization of vessel features extracted from a dataset of 1,800 samples comprising 3 classes of vessels sourced from the database. (B) Confusion matrix for classification tests on the test dataset sourced from the database (3 classes, 270 samples). Each row and each column represent an instance of a predicted class and a true class, respectively. Diagonal values indicate correct predictions. Green represents the accuracy of the predictions. (C) Prediction results for target signals collected by CHAS (3 classes, 1,800 samples); the black line indicates the true class, and the asterisk denotes the predicted class. (D) Confusion matrix for classification tests on the CHAS test dataset (3 classes, 1,800 samples). Green represents the accuracy of the predictions.

After determining the appropriate number of samples and features, we proceeded with training the classification model. We started by visualizing in 3 dimensions the features of 1,800 samples derived from 3 types of vessel signals in the database. Figure 6A shows the 3-dimensional spatial distribution of these sample features. Each point represents the feature information for a particular type of vessel, and the clear delineation among the feature distributions demonstrates the effectiveness of the spectrogram feature extraction method employed in this study. Finally, we trained the MLP model. Figure 6B displays the confusion matrix for the test dataset, comparing the predicted results with the true labels. Each row represents a true category, and each column represents a predicted category, with diagonal values indicating correct predictions. The results show that the model achieves strong classification performance, with an overall accuracy of 96.3%. Specifically, the classification accuracy for samples of the first class is 100%. For samples of the third class, some overlap with the other 2 classes results in a few misclassifications, leading to a slightly lower accuracy of 92.9%. We also compared other machine learning classification algorithms, such as *K*-nearest neighbors, Gaussian support vector machines, and the random forest bagged tree algorithm. The results showed that the MLP algorithm based on the BPNN had the highest classification accuracy (Fig. S4).

Finally, we evaluated the MLP classification model trained on the database using a separate test set composed of target signals collected by the sensor (Fig. 6C and D). Despite some confusion between categories, the model achieved an overall accuracy of 89.1% on CHAS-acquired signals, demonstrating that the sensor preserves the key acoustic features required for reliable classification under realistic noise conditions. Unlike most previous studies that applied learning algorithms only to standard datasets, the trained model was directly validated using signals acquired by CHAS, demonstrating effective transfer from database-trained models to real sensor measurements. The strong agreement validates the cross-domain consistency of the hydrogel-based sensing mechanism and highlights the proposed sensor’s potential for intelligent underwater perception systems.

In practical underwater acoustic applications, variations in hydrostatic pressure and temperature inevitably affect hydrophone performance. To address these challenges, future work will focus on enhancing the environmental adaptability of the sensor through material optimization and improved encapsulation design. Long-term reliability has already been verified by a 10-d durability test at 125 Hz, showing minimal sensitivity variation after 200 h of continuous operation (Fig. S5). Building on this, subsequent studies will investigate temperature-dependent stability and conduct systematic directivity measurements to clarify the spatial response behavior of the sensor in complex underwater environments. Moreover, efforts will be made to expand the detectable frequency range and develop multisensor array configurations for intelligent underwater sensing. These studies are expected to further improve the stability, precision, and applicability of CHAS in real oceanic conditions.

## Conclusion

In summary, this study demonstrates an integrated strategy for low-frequency underwater acoustic sensing that addresses the long-standing limitation of inefficient acoustic–mechanical–electrical coupling under weak pressure excitation. The proposed CHAS combines a micro-pyramid iontronic structure enabling enhanced EDL transduction with phase-sensitive lock-in amplification to preserve weak acoustic information across the 20 to 1,000 Hz range, achieving an average sensitivity of −158.86 dB. Beyond sensitivity enhancement, this work shows that sensor design plays a decisive role in enabling reliable intelligent acoustic analysis. Signals acquired by the sensor retain physically meaningful acoustic features, enabling the MLP-based classification framework (trained solely on the ShipsEar database with 96.3% accuracy) to directly achieve 89.1% accuracy on CHAS measurements without any retraining. Overall, this work advances hydrogel acoustic sensors from material-level optimization toward device–circuit–algorithm co-designed intelligent sensing systems, establishing iontronic hydrogel sensors as reliable front-end platforms for intelligent low-frequency underwater acoustic monitoring. Future work will focus on extending operational bandwidth, improving long-term stability, and developing distributed sensor arrays for practical ocean deployment.

## Materials and Methods

### Materials

AAm, *N*,*N*′-methylenebis(acrylamide) (MBAA), ammonium persulfate (APS), *N*,*N*,*N*′,*N*′-tetramethylethylenediamine (TEMED), and lithium chloride (LiCl) were obtained from Aladdin. Latex films, with a thickness of 0.3 mm, were provided by Suzhou Tuoce Instrument Equipment Co., Ltd. The Si mold was sourced and processed by Yancheng Dejing Technology Co., Ltd.

### Preparation of the AAm hydrogel

To prepare the AAm hydrogel, first add AAm (2.5 g), MBAA (0.125 g), and APS (0.01 g) to deionized water (10 ml). Then, dissolve LiCl (1 M) in the resulting solution. This mixture is subjected to a 5-min ultrasonic bath to ensure complete dissolution of the components. Afterward, introduce TEMED (2 μl) to initiate the polymerization process. Pour the resulting solution into a mold with a 4-inch diameter and a pyramid-shaped microstructure, as shown in Fig. S6. Finally, cure the polymerized solution at 50 °C for 1 h to obtain the AAm hydrogel.

The mold preparation involves using a silicon wafer of ⟨100⟩ orientation, coated with a 100-nm-thick layer of silicon nitride. This wafer undergoes photolithography and reactive ion etching for patterning, followed by potassium hydroxide (KOH) etching to create pyramid-shaped structures with a sidewall angle of 54.7°. After etching, the oxide layer is removed with buffered hydrofluoric acid, and trichloro(1*H*,1*H*,2*H*,2*H*-perfluorooctyl)silane is applied to aid in demolding.

### Fabrication of CHAS

A latex film is affixed to a stainless steel ring and coated on its inner side with conductive silver paint and then allowed to dry for 2 h. Next, a 550-μm-thick AAm hydrogel is cut into 15-mm-diameter discs. The hydrogel film is placed onto the lower electrode, with a 550-μm insulating gasket around the edges to prevent electrical conduction between the upper and lower electrodes. The latex film with the silver electrode is then attached to the base of the lower electrode and mounted onto a backing plate. The entire assembly is enclosed in an aluminum alloy casing and sealed with an O-ring for waterproofing (Fig. S7). The sensor is connected to an external conditioning circuit through a waterproof cable.

### Characterization and measurement

The underwater acoustic measurement setup consists of several key components: a signal generator (RIGOL DG822, with a 25-MHz bandwidth and a maximum sampling rate of 125 MSa/s), a power amplifier (Aigtek ATA-304, providing a maximum output voltage of 90 V_p-p_ and a bandwidth from dc to 30 kHz), and an underwater transducer (model no. UWS-110, with a frequency range of 80 to 22 kHz). A standard hydrophone (BK (8104), frequency range 0.1 Hz to 120 kHz) was used as the reference. According to its calibration certificate, the bare sensitivity of the hydrophone is −205 dB re 1 V/μPa. The hydrophone was connected to a BK BP 1702 conditioning amplifier (the recommended accessory), which provides adjustable sensitivity settings. In our measurements, the system sensitivity was set to 10 mV/Pa, corresponding to −160 dB re 1 V/μPa. An oscilloscope (Tektronix MSO54, with a 350-MHz bandwidth and a maximum sampling rate of 6.25 GS/s). The experiments were conducted in a 1 m × 1 m × 1 m acrylic water tank with a water area of 0.95 m × 0.95 m × 0.4 m. The signal generator produces single-frequency sinusoidal signals, which are amplified before being fed to the underwater transducer. The signal generator also provides the conditioning circuit with an ac excitation source (0.2 V, 40 kHz) and supplies the phase-locked module with a 40-kHz ac reference signal. Both the acoustic signal output from the conditioning circuit and the signal measured by the BK (8104) hydrophone are connected to the oscilloscope for storage. All acoustic sensors are placed 20 cm underwater along the sound axis, at a distance of 25 cm from the sound source.

## Data Availability

The datasets supporting the conclusions of this article are included within the article and its Supplementary Materials. Additional data, including raw sequencing data and experimental results, are available from the corresponding authors upon reasonable request.

## Supplementary Materials

Supplementary 1 Note S1 Figs. S1 to S7 Movie S1
