# Supplementary material for: High-Performance Iontronic Hydrogel Acoustic Sensor for Low-Frequency Underwater Sound Detection and Intelligent Recognition
Source: Research (Wash D C). 2026 May 28;9:1292. doi: 10.34133/research.1292 (PMC13216867; doi:10.34133/research.1292)
Supplement: Supplementary 1 — Note S1 Figs. S1 to S7 Movie S1 [file research.1292.f1292.zip › Supporting Materials.docx]

**Supplementary Information**

**High-Performance Iontronic Hydrogel Acoustic Sensor for Low-Frequency Underwater Sound Detection and Intelligent Recognition**

Jiawei Zhao^1,2*^, Honglei Zhou^1,2^, Tongqiang Fu^3^, Haijun Wang^3^, Tangjia Zhang^3^, Longqiu Li^1,2*^

^1^ Zhengzhou Advanced Research Institute of Harbin Institute of Technology, Zhengzhou 450000, China

^2^ State Key Laboratory of Robotics and System, Harbin Institute of Technology, Harbin 150001, China

^3^ School of Mechanical Engineering, Xi’an Jiaotong University, Xi’an 710049, China

^*^Address correspondence to: jw.zhao@stu.xjtu.edu.cn

E-mail: jw.zhao@stu.xjtu.edu.cn (J.Z.) and longqiuli@hit.edu.cn (L.L.)

**Supplementary Note 1：**

The thickness of the Electric Double Layer (EDL) is defined by the gap between the ionic layer and the opposite charge from the electrode, as shown in Equation (1).

$$K^{-1}=\frac{\varepsilon kT}{e^{2}\sum n_{i}z_{i}^{2}}$$

Where $K^{-1}$ is the EDL thickness or Debye length, $n_{i}$ is the ionic concentration (number of ions per unit volume), $z_{i}$ is the ionic valence, and e is the electron charge, $\varepsilon$ is the permittivity (dielectric constant) of solution, $k$ is Boltzmann constant, $T$ is the thermodynamic temperature.

$$C=\frac{\varepsilon_{0}\varepsilon A}{d}$$

Where $C$ is the capacitance of parallel plate capacitor, $\varepsilon_{0}$ is the vacuum dielectric constant, $\varepsilon$ is the dielectric constant of the intermediate medium; $A$ is the area of two electrode plates; $d$ is the distance between the electrodes.

According to Equation (2), the capacitance of a hydrogel sensor increases when the contact area 𝐴 between the electrode and the gel increases, or when the distance 𝑑 between the electrodes decreases. This indicates that creating hydrogel materials with dendritic electrode interfaces is beneficial.

Substitute Equation (1) into Equation (2):

$$C=\frac{\varepsilon_{0}e^{2}\sum n_{i}z_{i}^{2}}{kT}A=C_{u}*A$$

Here, $C_{u}$ represents the constant (capacitance per unit area), suggesting that *C* is an inherent property of the material. Consequently, once the dielectric composition is fixed, the EDL capacitance$C$is mainly determined by the contact area between the gel and electrode$A$.

**Supplement Figures：**


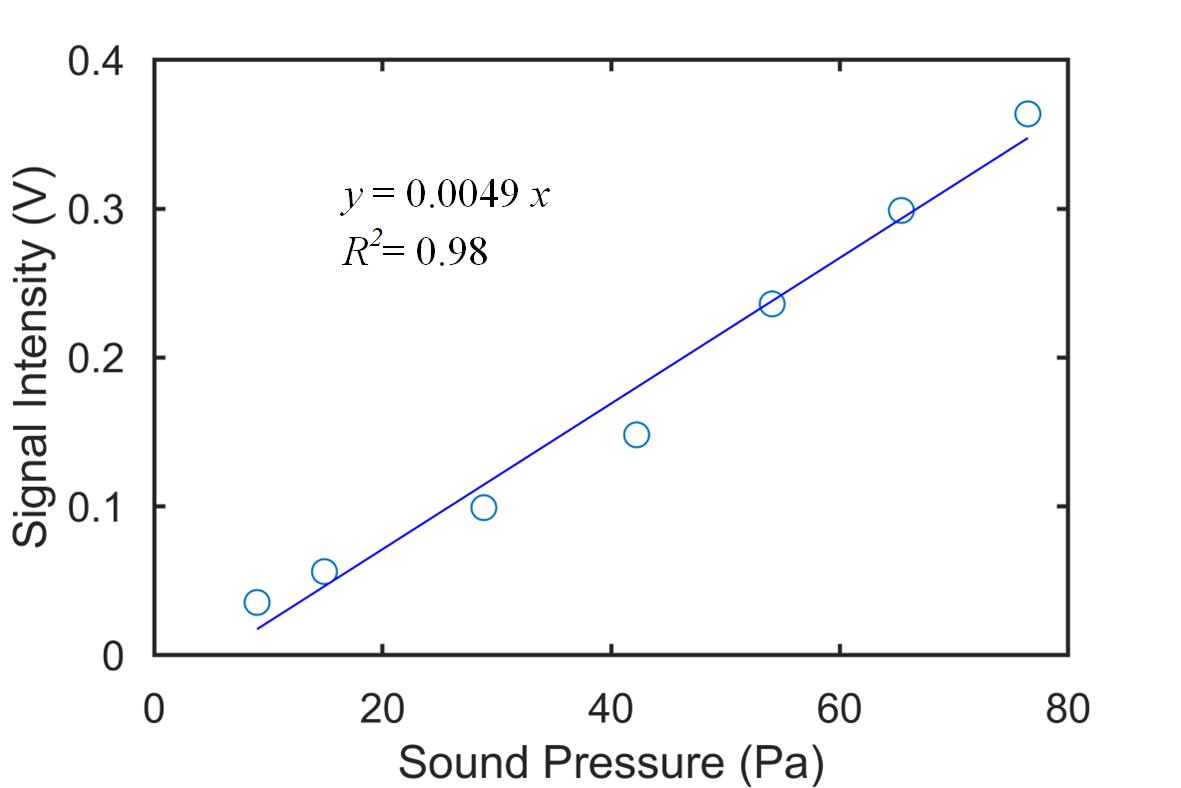


**Figure S1.** The corresponding fitting line between the output voltage and the input sound pressure at 200 Hz.


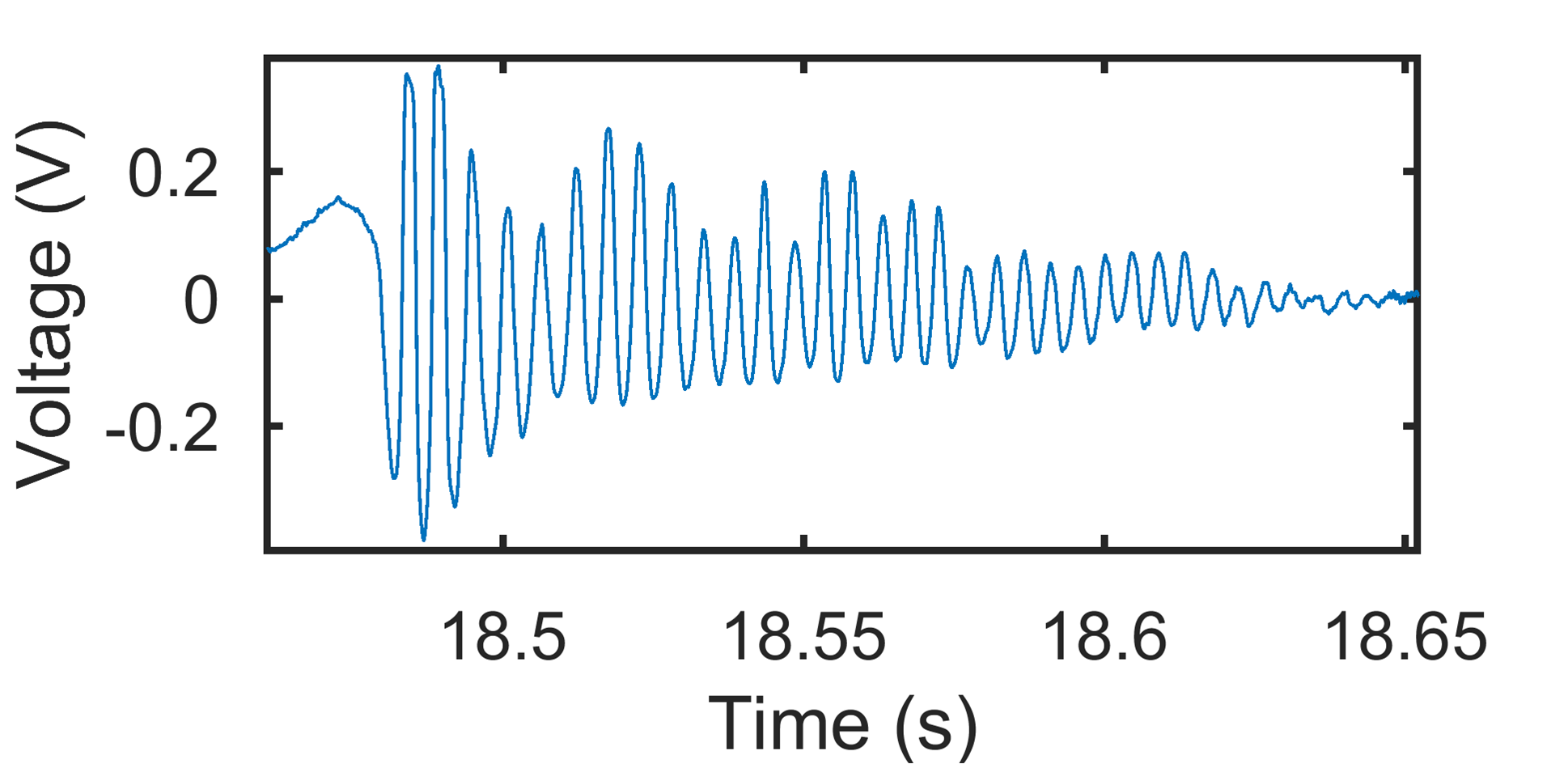


**Figure S2.** The enlarged voltage waveform with a maximum of 745mV.


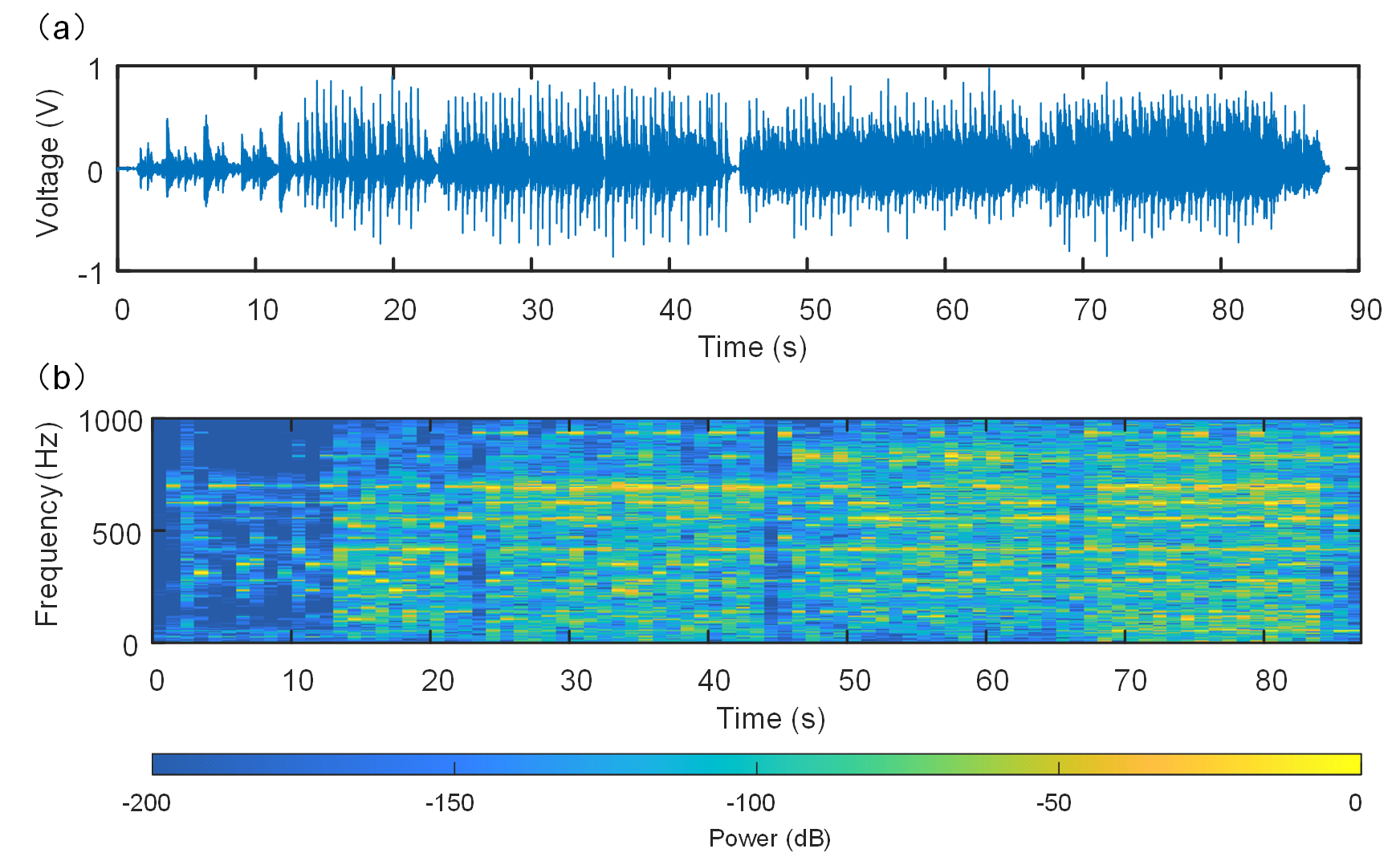


**Figure S3.** The time-domain waveform (a) and spectrogram (b) of the music 'China-E' recorded by CHAS.


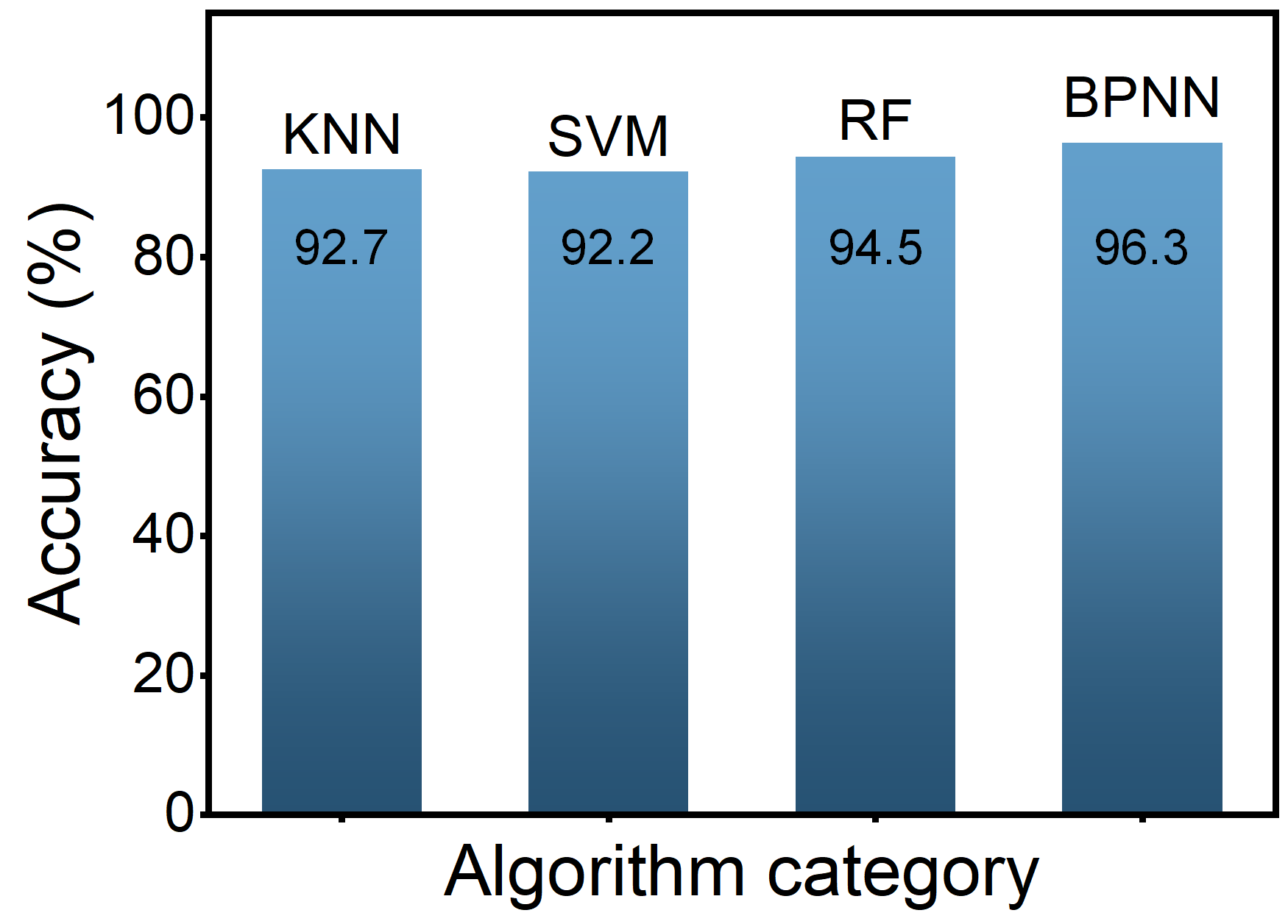


**Figure S4.** Comparison of classification results of different machine learning classification algorithms on the same feature set.


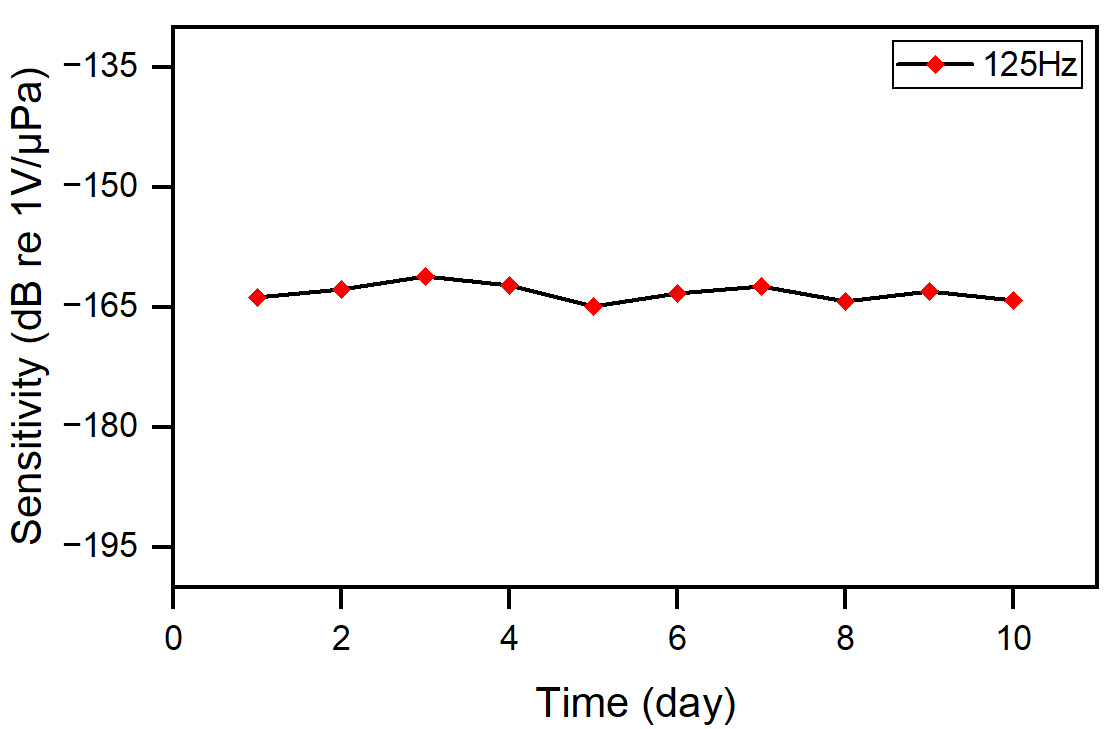


**Figure S5.** Durability test result of CH at 125Hz.


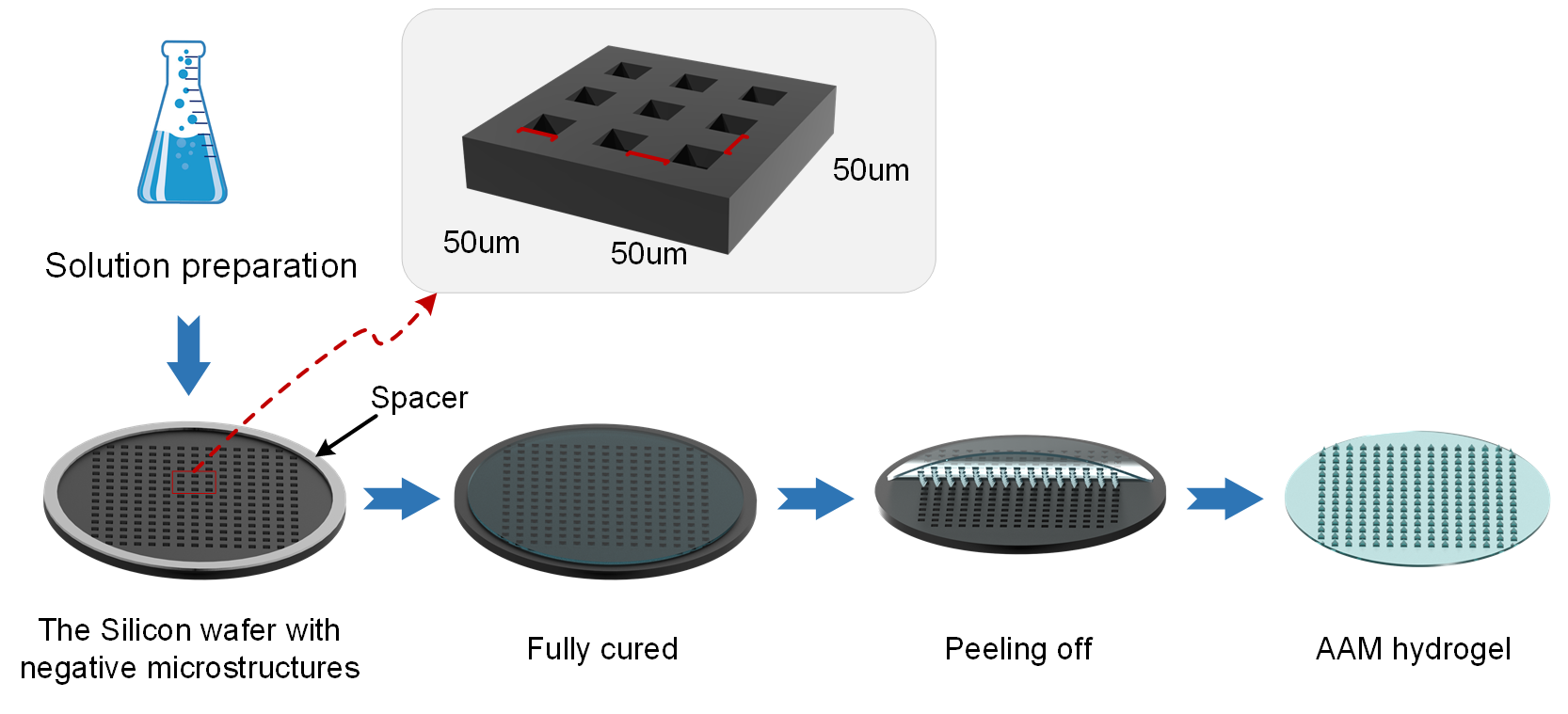


**Figure S6.** Schematic process for AAm hydrogel fabrication.


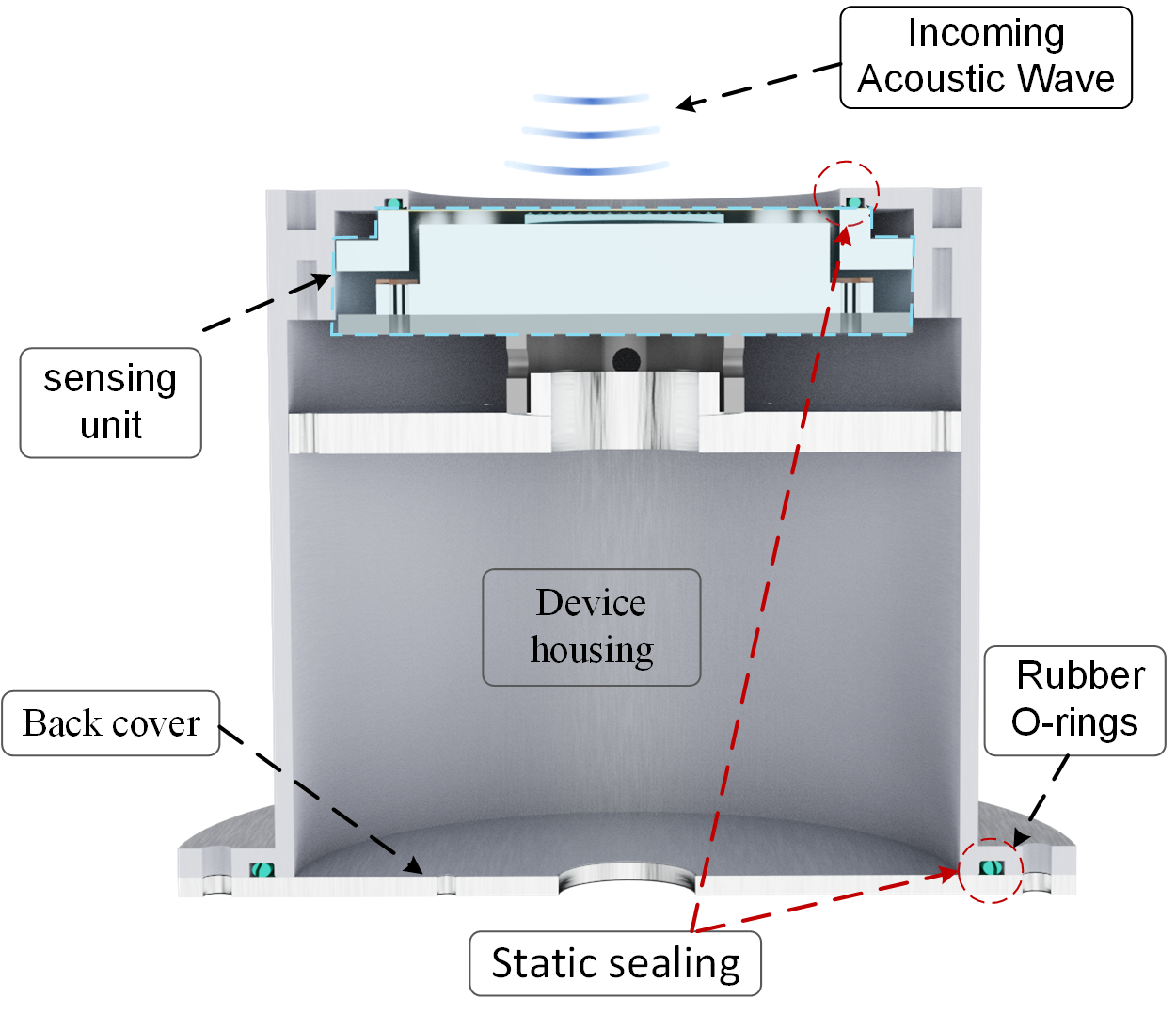


**Figure S7.** Schematic diagram of waterproof static sealing for CHAS.

Movie S1: A piece of music titled "China-E" recorded by CHAS.
